# Extracellular Vesicles in Renal Inflammatory Diseases: Revealing Mechanisms of Extracellular Vesicle-Mediated Macrophage Regulation

**DOI:** 10.3390/ijms26083646

**Published:** 2025-04-12

**Authors:** Jiatai Wei, Zijie Xie, Xiaodong Kuang

**Affiliations:** 1 The Second Clinical Medical College, Nanchang University, Nanchang 330031, China; 19318589176@163.com (J.W.); 19189479691@163.com (Z.X.); 2 Pathology Teaching and Research Office, School of Basic Medical Sciences, Jiangxi Medical College, Nanchang University, Nanchang 330031, China

**Keywords:** extracellular vesicles, macrophages, renal inflammatory disease, acute kidney injury (AKI), chronic kidney disease (CKD)

## Abstract

Renal inflammatory diseases are a group of severe conditions marked by significant morbidity and mortality. Extracellular vesicles (EVs), as facilitators of intercellular communication, have been recognized as pivotal regulators of renal inflammatory diseases, significantly contributing to these conditions by modulating immune responses among other mechanisms. This review highlights the intricate mechanisms through which EVs modulate macrophage-kidney cell interactions by regulating macrophages, the principal immune cells within the renal milieu. This regulation subsequently influences the pathophysiology of renal inflammatory diseases such as acute kidney injury and chronic kidney disease. Furthermore, understanding these mechanisms offers novel opportunities to alleviate the severe consequences associated with renal inflammatory diseases. In addition, we summarize the therapeutic landscape based on EV-mediated macrophage regulatory mechanisms, highlighting the potential of EVs as biomarkers and therapeutic targets as well as the challenges and limitations of translating therapies into clinical practice.

## 1. Introduction

Renal inflammatory disease represents a significant global health issue and increases the risk of several leading causes of death worldwide, affecting an estimated 10% of the global population [1,2]. The characteristics of these conditions are the proliferation of tubular epithelial cells (TECs) and mesangial cells, thickening of the basement membrane, podocyte apoptosis, tubulointerstitial fibrosis, and the infiltration of inflammatory cells [3,4]. The most prevalent forms of renal inflammatory diseases are acute kidney injury (AKI) and chronic kidney disease (CKD) [5]. Macrophages, abundant renal immune cells, undergo phenotypic and functional transformation during kidney injury. Initially, the M1 phenotype predominates, driving the inflammatory response [6], transitioning to the M2 phenotype later, which helps in the repair of epithelial and vascular endothelial cells [7].

Extracellular vesicles (EVs) are a group of small membrane particles released by cells into the extracellular space, consisting of proteins, lipids, and nucleic acids [8]. EVs can be used as biologically active carriers to carry a variety of goods, with proteins (including various receptors and signaling molecules) and nucleic acids embedded on their surfaces, which can participate in complex intercellular communication, molecular transfer, and gene expression [9]. Extracellular vesicles can act as tools and targets in therapy for diseases. EVs can be classified into apoptotic bodies, microvesicles, and exosomes based on their sources, characteristics, and release mechanisms. Apoptotic bodies are a byproduct of programmed cell death, released by encapsulating cell fragments during apoptosis [10]. Considering their limited application in disease models, apoptotic bodies will not be considered here [11]. The last two types of vesicles are most worthy of in-depth research due to their biological characteristics, important physiological functions, and clinical therapeutic significance [12]. Microvesicles originate from the plasma membrane, ranging in size from 50 to 1000 nm, and are secreted directly through extracellular phagocytosis without involving the cell membrane [13]. Exosomes are nanoscale extracellular vesicles, typically measuring 40–100 nm in diameter [14], and encapsulated by a lipid bilayer [15]. They come from various living cells [16] and are stable in several body fluids, including urine, prostate fluid, semen, and bile [17]. Exosomes transport a broad range of biological macromolecules [18] such as RNAs and proteins, modulating the pathophysiology of recipient cells by releasing these molecules into the extracellular space, initiating signaling and downstream transport [19]. As intercellular signaling mediators, exosomes play a crucial role in communication between macrophages and kidney cells [20,21,22]. Due to the use of EVs to study the pathophysiological mechanisms of renal inflammation, efforts have been focused on exosomes as biomarkers and signaling molecule carriers for therapeutic applications. In this review, we will particularly emphasize exosomes. However, the exact differences between these different vesicle groups are still controversial, so in appropriate circumstances, EV names are preferred. Based on this, we discussed the interaction between macrophages and kidney cells in the occurrence and development of renal inflammatory diseases, the mechanism of EV regulation, and the potential application of EVs as therapeutic targets.

## 2. Macrophages and EVs in the Kidney

### 2.1. Origin of Macrophages in the Kidney

Macrophages, key innate immune cells with phagocytic capabilities, are vital for maintaining renal homeostasis [7,14,15]. There are three primary origins of resident kidney macrophages: (1) yolk sac erythrocytic myeloid progenitor cells (EMP), (2) fetal liver EMP, and (3) hematopoietic stem cells (HSCs). The distribution of each progenitor cell type varies with developmental changes and the health of the kidney [23].

### 2.2. Plasticity and Polarization of Macrophages in the Kidney

Macrophages are distinguished by their capability to divide into two subpopulations, M1 and M2, based on their origin, surface markers, and secreted cytokines. Classical M1-type macrophages, induced by Th1-type cytokines, secrete pro-inflammatory cytokines such as IL-1β and TNF-α, exacerbating the inflammatory response. Conversely, alternative M2-type macrophages, induced by Th2-type cytokines, possess significant scavenging capacity and secrete cytokines like IL10 and TGF-β, which mitigate the inflammatory response and aid in tissue repair [10]. Initially, kidney inflammation is dominated by M1-type macrophages. However, as inflammation progresses, M2-type macrophages become more prevalent, playing anti-inflammatory and pro-repair roles [24,25]. Prolonged chronic kidney inflammation leads to an accumulation of M1/M2 macrophages and an increase in extracellular matrix (ECM) synthesis, aggravating kidney fibrosis. Recent studies have further subdivided M2-type macrophages into M2a, M2b, and M2c subgroups [26]. M2a macrophages promote Th2-like anti-inflammatory immune responses and tissue fibrosis; M2b macrophages are involved in immune regulation following Th2-like activation; and M2c macrophages play a significant anti-inflammatory role, contributing to immunosuppression, matrix deposition, and tissue remodeling [6,26,27].

### 2.3. EVs and Kidney Disease

**Figure 1:**
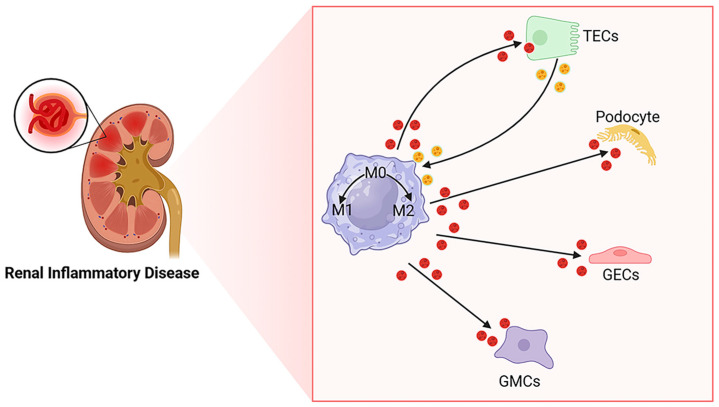
EVs mediate crosstalk between macrophages and other cells. EVs play a role in renal inflammatory diseases by mediating communication between macrophages and renal cells (podocytes, glomerular endothelial cells (GECs), TECs, and glomerular mesangial cells (GMCs)).

Recent research has increasingly focused on the mechanisms, prognostic, and treatment-related roles of EVs in kidney diseases [28]. Proteomic analysis shows that kidney-derived EVs primarily originate from glomerular podocytes and renal tubular cells of the proximal, distal, and collecting tubules [29], with macrophages also being a significant source [30]. Research has shown that EVs secreted by kidney cells can regulate macrophage polarization into M1 and M2 phenotypes, and in turn, the survival of kidney cells can be influenced by EVs released from macrophages (Figure 1). For instance, TECs may release albumin-stimulated EVs carrying CCL2 mRNA into macrophages, prompting polarization to the M1 type, which induces renal interstitial inflammation [31]. EVs derived from M1-type macrophages transport miR-155, targeting SOCS-1 to exacerbate renal tubular injury [32]. Similarly, EVs from other cells also play a role in renal pathology. For example, mesenchymal stem cells (MSCs)—pluripotent stem cells isolated from many tissues [33,34]—promote M2-type macrophage polarization and exert anti-inflammatory effects [35].

In conclusion, EVs are pivotal in maintaining kidney homeostasis by mediating intercellular communication and effectively transporting functional molecules between cells, thus impacting the behavior of recipient cells.

## 3. EV-Mediated Macrophage Regulation in AKI

AKI represents a clinical syndrome with significant mortality and morbidity [36,37,38], marked by renal failure leading to electrolyte–acid–base disorders, extracellular volume disorders, and accumulation of nitrogenous wastes [39]. Historically, AKIs were categorized into pre-renal, intrinsic, and post-renal types based on semi-anatomical associations with kidney structures. However, they are now classified into ischemia/reperfusion (I/R), nephrotoxic drugs, and sepsis-associated AKIs based on more specific syndromic descriptions [40,41].

### 3.1. Ischemia/Reperfusion AKI (I/R-AKI)

Kidney ischemia/reperfusion (IRI) is an important cause of AKI, triggering an inflammatory cascade that leads to high mortality [42,43,44]. The primary pathological features of I/R-AKI are tubular injury and immune cell infiltration [45]. The inflammatory response begins when kidney blood flow is transiently reduced or halted, followed by the re-establishment of perfusion. Thus, mitigating the inflammatory response is crucial in treating I/R-AKI [46].

At the onset of I/R-AKI, TECs not only suffer passive damage but also produce multiple chemokines and cytokines [47] that recruit M1-type macrophages to the kidney interstitium. These macrophages, upon recognizing damage-associated molecular patterns through their pattern recognition receptors (PRR), activate non-infectious inflammation, further worsening kidney damage [39]. This process results in a cycle of inflammation and kidney injury. During the repair phase, TECs may induce a switch in macrophages to the M2-type, which aids in clearing inflammation and repairing renal tubules [48,49], highlighting the key in TEC-macrophage crosstalk in the development of AKI.

When I/R-AKI occurs, macrophage-derived EVs facilitate crosstalk between macrophages and kidney cells, regulating kidney injury. EVs from macrophages in mice with AKI carry miR-195a-5p, which impairs mitochondrial function and induces damage in TECs, exacerbating AKI [50]. Similarly, TECs can produce EVs that influence macrophage polarization. EVs from damaged TECs transfer miR-374b-5p and miR-19b-3p to macrophages, triggering M1-type activation and increasing the release of inflammatory factors [22,51]. Additionally, endoplasmic reticulum (ER) stress is associated with the severity of I/R-AKI. EVs from ER-stressed TECs delivered to macrophages carry miR-106b-5p, which induces the polarization of macrophages into the M1-type and stimulates the development of renal histological lesions by suppressing ATL3 expression [52]. Liu et al. constructed an IR mouse model and found that HIF-1α induces hypoxia-impaired TECs to deliver EVs containing miRNA-23a to macrophages, enhancing M1 polarization and exacerbating inflammatory responses by inhibiting the ubiquitin editing protein A20 [19].

### 3.2. Sepsis-Induced AKI (S-AKI)

Sepsis, manifested as an inflammatory response syndrome with organ dysfunction and immune disorders following bacterial infection [53], frequently leads to AKI, a major complication significantly increasing mortality in septic patients [54]. There are multiple complex mechanisms behind S-AKI [55,56,57], with an over-activated inflammatory response emerging as a key factor [58,59].

In the initial stages of sepsis, macrophages migrate to infected tissues, responding to toll-like receptor signals activated by LPS and damaged TECs and becoming polarized to the M1-type. They upregulate the expression of inflammatory mediators [60], regulated by nuclear transcription factor-κB (NF-κB) [61,62,63]. These mediators further promote NF-kB activation, escalating the inflammatory response [64,65,66,67]. In later stages, TECs encourage a shift to the M2 macrophage phenotype by secreting colony-stimulating factor 2 (Csf2) and activating p-STAT5 [68]. M2-type macrophages then mitigate kidney injury by decreasing apoptosis in proximal TECs of the renal cortex through elevated IL-10 expression and reduced TNF-α secretion [69].

The role of EVs in S-AKI has now been confirmed. In Zhang et al.’s experiment, EVs secreted by human umbilical cord MSCs (hucMSCs) are able to alleviate S-AKI symptoms by reducing IRAK1 expression and suppressing NF-κB activity via the upregulation of miR-146b [70]. Platelet-derived EVs exacerbate the release of inflammatory mediators, apoptosis, and oxidative stress in TECs by transferring ARF6 and targeting the ERK/Smad3/p53 pathway [71]. EVs carrying miR-19b-3p from TECs have been found to induce the polarization of M1-type macrophages [22], and CCL2-containing EVs from TECs exacerbate S-AKI by promoting macrophage infiltration and pro-inflammatory cytokine expression [63]. Pyroptosis, a recently identified form of cell death [72], occurs in macrophages and TECs and is considered a basis for S-AKI [73,74,75,76]. CircMLH3 in plasma-derived EVs promotes macrophage pyroptosis by sponging miR-590-3p and regulating TAK1 expression, triggering a severe cytokine storm [77]. By establishing a mouse model of LPS-induced AKI, Xiong et al. demonstrated that acid sphingomyelinase (ASM) promotes EVs secretion by macrophages, and these Exos are internalized by surrounding endothelial cells, inducing damage to GECs, whereas amitriptyline, an ASM inhibitor, reduces the release of EXOs after LPS stimulation and inhibited endothelial cell damage by EVs, which provides a new target for S-AKI treatment [78].

Monitoring EVs from macrophages, linked closely with disease progression, could aid in the early diagnosis and treatment of S-AKI. Chen et al. found that M1-type macrophage-derived EV miR-93-5p enhances pyroptosis in TECs by binding to TXNIP, while M2-type EVs protect TECs from damage and preserve their viability [79]. Sirtuin 1 (SIRT1), a highly conserved mammalian NAD+-dependent histone deacetylase, has been reported to inhibit the production of pro-inflammatory cytokines in macrophages and is associated with anti-inflammatory protection. However, the excessive activation of SIRT1 may promote fibrosis by M2 macrophages secreting TGF-β [80,81]. A study confirmed that miR-138 expression is elevated in LPS-stimulated macrophages in an animal model of LPS-induced inflammation. This elevation exacerbates sepsis-induced renal injury by targeting SIRT1, inhibiting the NF-κB pathway, and promoting M1 macrophage polarization [82]. Moreover, Cao et al. found that miR-9 promotes sepsis-induced kidney injury by targeting SIRT1 in macrophages [83]. In another study, EVs from endothelial progenitor cells encapsulated TUG1 to stimulate M2 macrophage polarization and inhibited inflammation by suppressing miR-9-5p to upregulate SIRT1 expression, which prevented the development of sepsis and alleviated sepsis-induced renal damage [84].

### 3.3. Cisplatin-Induced AKI (CI-AKI)

Cisplatin, a heavy metal-based chemotherapeutic agent, is widely utilized in cancer therapy but can cause severe nephrotoxic effects with long-term use [85]. The primary characteristics of CI-AKI are tubular necrosis and acute inflammation [86,87].

Cisplatin gathers in renal proximal tubular cells, promoting CI-AKI. Upon cisplatin entry into renal tubular cells, the cytochrome P450 enzyme system on the ER membrane is activated, inducing oxidative stress-promoted toxicity and activating caspase-12-mediated cell death [88]. Additionally, cisplatin causes DNA damage and produces factors, inducing p53 production and stimulating the Bax/Bcl-2 pathway in mitochondria. This activates pro-apoptotic genes caspase-9 and caspase-3, resulting in TEC death [89]. The dead TECs then secrete large amounts of pro-inflammatory cytokines (IL-34, SAP130, and CXCL16), triggering cytokine expression and recruiting and polarizing M1-type macrophages [89,90], further exacerbating kidney injury [91]. In the later stages of CI-AKI, cisplatin damage to TECs induces M2-type macrophage polarization, which promotes epithelial–mesenchymal transition (EMT) in TECs [92].

**Figure 2:**
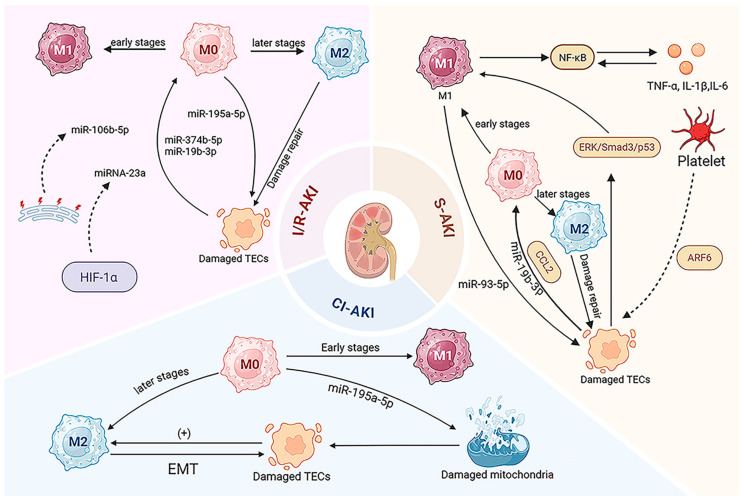
The regulatory mechanism of EVs in AKI. The crosstalk mediated by EVs between macrophages and renal cells in the kidneys significantly contributes to the development of AKI. The different regions in the figure represent examples of this crosstalk in different acute kidney injuries.

Although macrophage and TEC crosstalk is critical in CI-AKI, few studies have focused on EVs [93], mainly examining macrophage interactions with mitochondria. Mitochondrial metabolism in TECs, heavily influenced by their dense distribution, plays a crucial role in TEC survival [94]. For instance, mitochondria undergo autophagy to reduce cisplatin-induced TEC death via the ROS/HO-1/GPX4 axis [95]. If damaged mitochondria are not cleared promptly, ROS are released, causing oxidative stress [96]. Macrophage-derived EVs miR-195a-5p can impair mitochondria in TECs, leading to an inflammatory response that worsens kidney injury [50,97,98] (Figure 2).

### 3.4. EVs as Diagnostic Biomarkers for AKI

Mediated macrophage regulation of EVs serves as an applicable diagnostic biomarker for AKI patients while participating in the progression of AKI. Studies have shown that the expression of urinary exosomal miR-19b-3p is positively correlated with the severity of albuminuria and the severity of renal tubulointerstitial inflammation, suggesting that miR-19b-3p may serve as a potential biomarker for renal disease [22]. In addition, the data showed that urinary exosomal mir-106b-5p levels were positively correlated with the degree of renal injury, suggesting that miR-106b-5p may serve as a suitable diagnostic biomarker for patients with AKI [52]. In another study, circMLH3 was found to be upregulated in the blood of patients with sepsis by analyzing the peripheral blood of eight patients with sepsis and nine patients with septic shock, demonstrating the predictive value of circMLH3 for the diagnosis and prognosis of sepsis-induced kidney injury [77].

### 3.5. AKI Therapeutic Strategies Targeting EV-Regulated Macrophages

Despite significant advances, the incidence and deterioration of AKI remain high due to its complex pathogenesis and the scarcity of early diagnosis and treatment methods [32,99,100,101]. There is an urgent need for new therapeutic approaches to delay kidney injury and halt the progression of kidney failure. As key mediators of intercellular communication, EVs can alleviate AKI by reducing inflammation, decreasing kidney cell death, and enhancing repair through various mechanisms. Moreover, EVs are notably stable, easy to store, and can effectively overcome biological barriers [102,103], making them a promising therapeutic tool for AKI. We have summarized recent studies on using EVs as delivery vehicles to target macrophages for the treatment of AKI, while also covering the therapeutic research of EVs derived from macrophages in AKI (Table 1).

## 4. EV-Mediated Macrophage Regulation in CKD

CKD represents a significant health and economic burden worldwide [116], and is projected to become the fifth leading cause of life loss globally by 2040 [117,118]. The hallmark of CKD is the persistent loss of kidney function, with fibrosis being a common pathway for CKD induction and an important characteristic of the disease [119,120]. Various factors can induce the occurrence of CKD, including diabetes mellitus, autoimmune diseases, and other conditions [121].

### 4.1. Kidney Fibrosis

Kidney fibrosis is a pathological state resulting from endogenous or exogenous pathogenic stimuli, characterized by ECM deposition [122]. Macrophages are crucial in fibrosis within the kidneys, contributing to the progression of kidney fibrosis by damaging kidney parenchyma and worsening kidney function.

Persistent and excessive inflammation has been shown to drive fibrosis [123]. At the onset of injury, M1-type macrophages are primarily involved, exacerbating inflammation by secreting pro-inflammatory factors and promoting myofibroblast proliferation and fibroblast recruitment [124]. These macrophages activate matrix metalloproteinase gelatinase A (MMP-2), facilitating the catabolism of ECM and promoting the EMT in TECs [125]. Furthermore, the overexpression of circACTR2 in M1 macrophages not only triggers NLRP3-induced macrophage pyroptosis and inflammation but also enhances fibrosis and EMT in TECs through the paracrine secretion of IL-1β [126]. Conversely, M2-type macrophages release various factors to stimulate myofibroblast proliferation and promote ECM accumulation [127].

**Figure 3:**
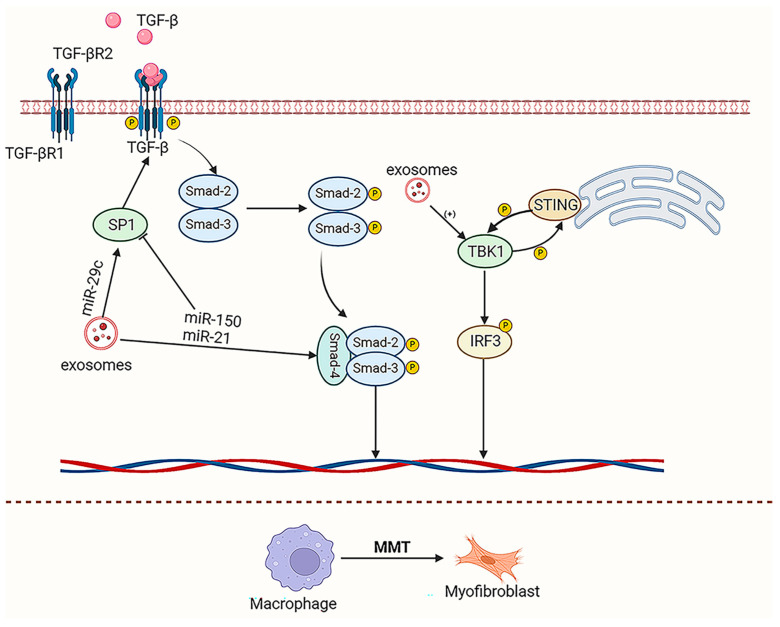
The role of EVs in macrophage-to-myofibroblast transformation (MMT) in kidney fibrosis. MMT is pivotal in kidney fibrosis through mechanisms such as TGF-β1/Smad3 and STING/TBK1. EVs can promote MMT-mediated tissue fibrosis by targeting the TGF-β/Smad3 pathway, or lead to a significant increase in α-SMA by activating the STING/TBK1 pathway, contributing to M2-MMT.

In addition to inducing fibroblast recruitment, proliferation, and activation that indirectly promote fibrosis, macrophages can directly contribute to fibrosis through macrophage–myofibroblast transformation (MMT) [127,128]. After kidney injury onset, macrophages infiltrate the kidney and differentiate into M2-type macrophages in response to inflammation, which then transforms into myofibroblasts. This transformation, coupled with an increase in ECM synthesis, drives the progression of kidney fibrosis, known as MMT [128]. In the late stage of kidney injury, M2-type macrophages predominantly promote the repair of epithelial and vascular endothelial cells. However, incomplete repair can trigger the transdifferentiation of M2-type macrophages, leading to fibrosis [7]. These macrophages stimulate myofibroblast proliferation through IL-10 and TGF-β secretion, contributing to late-stage fibrosis [129]. MMT plays a crucial role in renal fibrosis through various mechanisms, including the transforming growth factor-β 1 (TGF-β 1)/Smad3 signaling pathway and natural killer T cell (NKT)/IL-4 signaling pathway, as well as the adiponectin/APK pathway and ALD/MR/TGF-β 1 signaling pathway [130,131]. The levels of TGF-β signaling are crucial for inducing polarized M2-type macrophage subtypes. Superfluous TGF-β activation triggers MMT in M2a macrophages, TGF-β binds to the TGF-β receptor complex, phosphorylates the Smad family complex, and finally activates the Src-centered gene network in bone marrow-derived macrophages to promote MMT process. However, moderate levels of stimulation enhance the anti-inflammatory response associated with M2c macrophage differentiation. The regulation of the TGF-β levels and ATF6/TGF-β/Smad3 signaling axis in macrophages can direct their polarization towards the M2c phenotype [7]. Activated NKT cells produce excess IL-4, which then binds to IL-4 receptor alpha and triggers JAK3/STAT6 signaling to enhance myofibroblast transformation. Research has found that NETs formed by neutrophils stimulate the secretion of TNF-α and TGF-β 1 by macrophages by triggering the translocation of NF-κ B p65 subunit to the nucleus, and then promoting MMT by activating the TGF-β 1/Smad pathway in macrophages [132]. Under the regulation of extracellular vesicles, MMT plays a role in kidney fibrosis via TGF-β1/Smad3 and STING/TBK1 signaling pathways [130,131] (Figure 3).

EVs can promote fibrosis by inducing macrophage polarization, accelerating the processes of EMT and MMT. Yu et al. [133] found that extracellular vesicles derived from fibroblasts treated with TGF-β1 or Ctrl were administered to mice with FA-induced kidney injury. These extracellular vesicles enhanced the polarization of M2 macrophages via TBK1 signaling, thereby exacerbating renal fibrosis through MMT. In another study, researchers cloned circUBXN7 into a lentiviral vector and injected it into a DN mouse model, which resulted in the overexpression of extracellular vesicle circUBXN7 in TECs. This process forms a positive feedback loop with SP1 to recruit macrophages, leading to tubular EMT and fibrosis [134]. Furthermore, when EVs overexpressing miR-199a-5p were administered into the HFD/STZ mouse model through the tail vein, miR-199a-5p originating from TECs promoted the development of DN renal fibrosis by influencing the Klotho pathway and causing M1 macrophage polarization. Similarly, in another study, researchers first subjected TECs to EMT-induced treatment and subsequently injected EVs derived from TECs undergoing EMT treatment into a UUO-induced mouse model of renal fibrosis. They found that EVs derived from EMT-TECs triggered the activation of M1 macrophages, leading to further EMT and exacerbating renal fibrosis [135]. Urinary exosomal miR-21, miR-29c, and miR-150 promote the M2-MMT process and thereby renal fibrosis by targeting SP1 and the Smad3/TGFβ signaling pathway [136]. In diabetic nephropathy (DN), TEC-derived EV miRNA-34a promotes M1-type macrophage activation and induces tubular cell fibrosis through PPARGC1A gene expression [137]. Moreover, Qiang Zhong et al. [138] found that EVs from senescent cells contain miR-20a-5p and miR-21-5p, which activate the TGF-β/Smad pathway by downregulating smad7 expression and promoting smad3 phosphorylation. In addition, these EVs drive macrophages toward M2-like polarization and promote MMT through a positive feedback loop of miR-20a/miR-21-TGF-β. Meanwhile, TGF-β released by macrophages can stimulate renal tubular cells to produce exosomal miR-21, which facilitates fibroblast activation and encourages renal fibrosis by targeting PTEN [139]. The crosstalk between macrophages and neutrophils also plays a role in fibrosis. Neutrophil-derived EVs can affect renal fibrosis by inhibiting LPS-activated macrophages, decreasing their phagocytosis, maturation, and release of pro-inflammatory cytokines, while increasing TGF-β 1 excretion [140].

To more accurately demonstrate the role of EVs in the progression of renal fibrosis, Kim et al. demonstrated that exosomal miR-21 can activate NF-κB and induce inflammatory responses in TECs through TLR7 activation, thereby promoting renal fibrosis. Furthermore, they found that the knockdown of TLR7 in murine tubular epithelial cells effectively mitigated renal injury and fibrosis [139,141]. Xiao’s research revealed that TREM-2-/-macrophage-derived EVs facilitate interactions between macrophages and TECs via the HSPa1b/AKT pathway by silencing the triggering receptor expressed on myeloid cell-2 (TREM-2) in mouse macrophages, thereby modulating the crosstalk between macrophages and TECs to attenuate TEC fibrosis [142]. Additionally, the knockdown of exosomal let-7b-5p and miR-155 in a mouse model of renal fibrosis suppressed inflammation and alleviated renal fibrosis [143,144].

### 4.2. Diabetic Nephropathy (DN)

DN is a serious clinical complication affecting small blood vessels in diabetes mellitus patients and a leading contributor to CKD [2,145,146]. It is reported that approximately 30% of patients with type-1 diabetes and 40% with type-2 diabetes will develop DN, which is associated with high mortality [147]. The pathological features of DN include thickening of the glomerular basement membrane, podocyte apoptosis, tubulointerstitial fibrosis, and the recruitment and infiltration of inflammatory cells [148]. Complex metabolic disturbances contribute to the phenotypic transformation of macrophages in DN, with advanced glycation endproducts (AGEs), excessive adipose, and high glucose accumulation promoting the secretion of cytokines, adhesion molecules, and chemokines by kidney podocytes and mesangial cells, which activate macrophages and induce their transformation to a pro-inflammatory phenotype [135,149,150,151].

Crosstalk between macrophages and other cells contributes to DN, with EVs playing a role in this process:

(1) Crosstalk between macrophages and epithelial/tubular cells includes several mechanisms: Human serum albumin (HSA)-induced EVs miR-199a-5p from TECs is reported to induce kidney fibrosis and inflammation by polarizing M1-type macrophages and accelerating DN progression by targeting the Klotho/TLR4 signaling pathway [152], or by stabilizing HIF-1α to promote glycolysis in M1 macrophages [153]. EV miRNA-34a induces tubular cell fibrosis by negatively regulating PPARGC1A expression, promoting M1-type macrophage activation in DN [137]. Epsin1 induces the release of TEC-derived EVs Dll4 under hyperglycemic conditions, accelerating inflammation by encouraging macrophages to shift towards a pro-inflammatory state [15]. EVs derived from lipophilic TECs rich in LRG1 activate macrophages through the TGF β R1 dependent pathway, inducing inflammation in DN; at the same time, macrophages also feedback increase the expression of TRAIL, promoting the apoptosis of TECs [154]. Li et al. found that M1-type macrophage-derived EVs modify PAQR3 m6A through the secretion of METTL14, promoting high glucose-induced apoptosis in GECs and exacerbating DN-induced kidney injury [155].

(2) Crosstalk between macrophages and mesangial cells under high glucose conditions promotes the secretion of inflammatory cytokines and ECM [156]. Liu et al. discovered that exocytosis from high glucose-treated M1-type macrophages can activate NLRP3 inflammasomes and autophagy deficiency in mesangial cells, accelerating kidney injury [157], or activate mesangial cells by targeting the TGF-β1/Smad3 pathway, inducing basement membrane thickening and mesangial matrix expansion [158].

(3) In DN, macrophages are conclusively shown to promote podocyte injury and apoptosis. In the early stages of DN, the functional and structural impairment of podocytes leads to podocyte apoptosis and detachment, resulting in disruption of the integrity of the glomerular filtration barrier [159]. It has been reported that more than 20% depletion of podocytes can cause impairment of glomerular function. As glomerular disease progresses in diabetes, podocytes can be detected increasingly in the urine. This indicates that the loss of podocytes in this condition is linked to the advancement of glomerular disease [160]. Therefore, investigating podocyte depletion provides strong support for understanding the pathogenesis of DN. In DN, the macrophage-derived EVs miR-21-5p promotes podocyte injury by enhancing pyroptosis through tumor necrosis factor α-inducible protein 3 [161]. Zhuang found that EVs secreted by M1-type macrophages reduce cell proliferation by upregulating miR-21a-5p targeting the Tnpo1 gene and increasing apoptosis, thereby exacerbating high glucose-induced podocyte injury [162].

### 4.3. Lupus Nephritis (LN)

**Figure 4:**
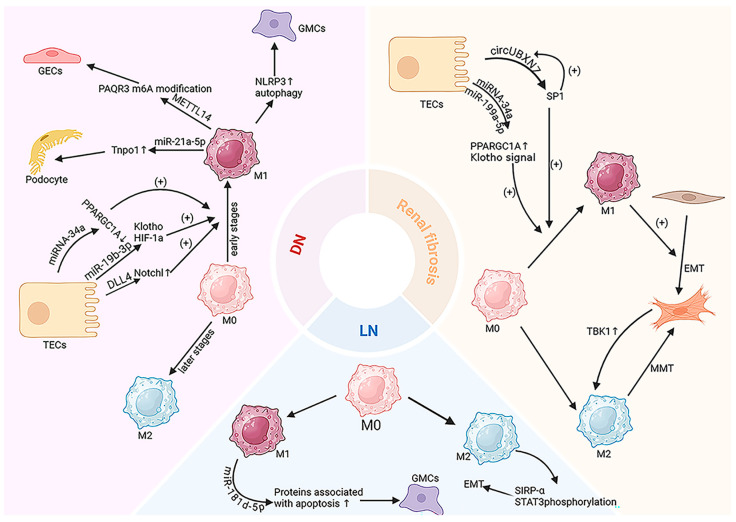
The regulatory mechanism of EVs in CKD. EV-mediated crosstalk between macrophages and renal non-myeloid cells contributes to the progression of chronic kidney disease. The different regions in the figure represent examples of this crosstalk in different chronic kidney diseases.

Lupus nephritis (LN) is a secondary nephritis resulting from systemic lupus erythematosus (SLE), affecting approximately half of the patients [163]. Macrophages are prevalent in the inflammatory tissue lesions of LN [164]. In the early stages of kidney injury, tubular cells trigger LN by producing large quantities of colony-stimulating factor that induces M1-type macrophage polarization, worsening repair dysfunction and the inflammatory response [165]. Crosstalk between macrophages and podocytes and mesangial cells is a crucial mechanism in LN development, disrupting the filter barrier and impairing kidney function through cytokine interactions [166,167]. EVs have provided a new direction for exploring the role of macrophages in LN, demonstrating that USC-Exo miR-21, miR-150, and miR-29c can exacerbate LN by enhancing the MMT process via the Smad3/TGFβ signaling pathway [136]. Additionally, EVs miR-382 has been shown to promote M2-type macrophage-induced kidney fibrosis by enhancing SIRP-α-mediated STAT3 phosphorylation [168]. Furthermore, macrophage-derived EVs miR-181d-5p disrupt the normal structure of mesangial cells by increasing proteins associated with mesangial cell pyroptosis [169] (Figure 4).

### 4.4. EVs as Diagnostic Biomarkers for CKD

EVs that mediate macrophage regulation have been identified as diagnostic biomarkers for CKD and are implicated in its progression. A study involving 49 patients with DN and 54 healthy controls revealed that circUBXN7 levels were significantly elevated in the plasma of patients with DN, indicating its potential as an early diagnostic marker for DN [134]. In the urinary extracellular vesicles of patients with LN, miR-21 and miR-150 were significantly upregulated, whereas miR-29c was downregulated, and their expression was closely associated with kidney injury [136]. Another study found that the TREM-1/TREM-2 ratio was decreased in patients with CKD, and notably, this ratio was significantly lower in patients with moderate-to-severe fibrosis than in those with non-mild renal fibrosis, suggesting that the urinary TREM-1/TREM-2 ratio could serve as a potential biomarker for diagnosing renal fibrosis [142]. In the context of polycystic kidney disease, miR-30d-5p was significantly downregulated in urinary EVs and human PKD1 cystic kidney tissues of patients, suggesting its potential as a novel biomarker for disease progression [135].

### 4.5. CKD Therapeutic Strategies Targeting EV-Regulated Macrophages

EV research focusing on their role as delivery vehicles targeting macrophages for CKD treatment was also reviewed, including therapeutic studies of macrophage-derived EVs, offering valuable insights into CKD treatment (Table 2).

## 5. Conclusions

This review explores the critical role of EV-mediated macrophage regulation in renal inflammatory diseases, where EVs act as carriers of pro- and anti-inflammatory signals. The complex kidney disease-mediated crosstalk between macrophages and renal cells emphasizes the intricacies of cellular communication and immune regulation in the context of inflammatory diseases of the kidney. This crosstalk is important for regulating (promoting/suppressing) various pathological states. Consequently, numerous targeted therapeutic strategies are being investigated, presenting novel opportunities for treating kidney inflammatory diseases. These strategies involve modulating EV release or changing the composition of their contents, such as employing EV inhibitors, creating EVs loaded with therapeutic agents, or altering the mechanism by which cells take up EVs.

The key to optimizing the treatment of renal inflammatory diseases lies in diagnostic and prognostic biomarkers. Traditional kidney biopsy carries the potential risk of serious postoperative complications such as infection and bleeding, and is not convenient for repeated evaluation of kidney injury. At the same time, some traditional biomarkers such as serum creatinine concentration, urinary protein concentration, etc., show low sensitivity in the early stages of kidney injury and are easily influenced by unrelated variables [181,182,183]. Therefore, the search for new and more sensitive non-invasive diagnostic biomarkers has become a vibrant field. Normal urine contains EVs from various epithelial cells in the renal environment, including endothelial cells, podocytes, renal tubular cells, collecting duct cells, and glomerular mesangial cells. Separating urine EVs can identify their source [184]. EVs isolated from urine exhibit RNA profiles similar to those of the kidneys, allowing us to monitor changes in overall kidney function through urine collection and analysis [185]. Many previous studies have also shown that EVs exhibit characteristic changes in both AKI and CKD, indicating their potential and substantial prospects as biomarkers in the field of renal inflammatory diseases. Firstly, EVs can serve as protein biomarkers. In critical care and surgical settings, elevated levels of EVs, which are markers of renal tubular injury (such as fetuin-A, NGAL, and uE-WT1), are associated with AKI/CKD, making it easier to track the location of lesions [186,187,188]. The presence of podocyte markers such as nephrin, podocalyxin [189,190], and podocin [191] determines an increase in EVs derived from podocytes or endothelium, indicating podocyte injury. The inflammatory factors such as TNF-α and IL-6 carried by EVs can also evaluate the degree of inflammation (such as LN) [192]. In addition to proteins and peptides, EVs also contain abundant mRNA and miRNA, which can reflect renal dysfunction and structural damage. For example, in patients with renal fibrosis, urinary exosomal miR-200b is elevated, and the decrease in miR-29c and CD2AP mRNA is associated with renal function and fibrosis severity [193]. The elevated expression level of WT1 mRNA in EVs of DN patients suggests the degree of glomerular injury [191,193,194,195,196]. In LN patients, urinary vessel miR-21, miR-29c, and miR-150 are potential predictive biomarkers for disease progression [197]. These findings collectively emphasize the crucial role of EV RNA as an important diagnostic and prognostic tool for renal inflammatory diseases (Table 3).

The potential of EVs as clinical biomarkers for diagnosis and prognosis has received increasing attention [209] due to several factors: (1) EVs are highly specific and can accurately reflect the physiopathological state of the cells involved, facilitating the early detection of disease for intervention; (2) compared with traditional biopsies, non-invasive or minimally invasive sampling methods of EVs reduce the patient’s adverse reactions and complication risk; (3) EVs are stable and detectable in body fluids, and are easy to store and transport in diagnosis; (4) EVs can reflect comprehensive body health information, which can help to provide more accurate and personalized treatment. In conclusion, exploring EVs as biomarkers provides a promising pathway to enhance our understanding of the molecular mechanisms of inflammatory diseases of the kidney and also lays the foundation for the development of new diagnostic and therapeutic strategies.

EVs have also opened up new fields for modern drug delivery. Currently, many materials such as liposomes, nanoparticles, hydrosols, etc., are being attempted to make delivery carriers, but they also face many challenges such as high systemic toxicity and low bioavailability. EV delivery solutions have multiple advantages [210,211]: (1) Safety—EVs, as endogenous carriers, have better tolerance, ensuring a safer and more effective drug delivery system. (2) Barrier penetrability—EVs, due to their small size, can penetrate multiple biological barriers. (3) Stability—EVs have a double-layer structure. (4) Specificity—Proteins on the surface of EVs can specifically target cells for delivery. The EV delivery scheme has been applied in many studies; for example, Kim et al. delivered the EV super repressor inhibitor of NF-κ B (Exo-srIkB) to mice through a novel optogenetic engineering EVs technology, reducing macrophage activation and improving I/R-AKI by lowering NF-kB signaling transduction [212]. EV delivery can also enhance drug properties. Yang et al. added the antibiotic linezolid to mouse RAW264.7 macrophage-derived EVs using incubation methods and found that the linezolid-encapsulated EVs had better inhibitory effects on intracellular methicillin-resistant Staphylococcus aureus infection without any signs of macrophage toxicity, which may be beneficial for the treatment of renal inflammation caused by microbial infections [213]. There is a study that added curcumin (an anti-inflammatory drug) to EVs derived from mouse tumor cell lines (EL-4). After various experiments, it was found that the anti-inflammatory activity of curcumin in EVs was increased, and inflammatory factors such as IL-6 and TNF-α were significantly reduced [214]. Traditional therapies for renal inflammatory diseases still face challenges such as inconsistent efficacy, high recurrence rates, and significant side effects, which cannot meet the treatment needs of patients. EVs have shown potential as alternatives to cell therapy in preclinical and clinical studies, with many data indicating the feasibility and safety of EV therapy. As mentioned earlier, EVs can serve as therapeutic agents. EVs carrying RNA can selectively deliver their contents to specific target cells, correcting the process of dysfunction, which endows EVs with great potential as therapeutic agents. For example, injecting EVs Epn1 KD derived from TECs into DN mice can significantly reduce Dll4/Notch1 signaling communication between the kidneys and macrophages, thereby alleviating inflammation [21]. Injecting EVs derived from BMSCs into mice can inhibit core fucosylation (CF), thereby improving fibrosis by suppressing the MMT of macrophages [215]. Injecting miR13896 of hucMSC sEV into CI-AKI mice can promote the transformation of M2 macrophages, inhibit inflammatory factors, and improve kidney function [216].

The therapeutic potential of EVs is also expanding rapidly, but there are still several challenges in this field [149]: (1) While EVs are critical in renal inflammatory diseases, the specific regulatory mechanisms and action targets for the bidirectional regulation between EVs and macrophages have not been fully determined. Future studies should further explore this relationship to uncover potential signaling pathways and molecular mechanisms [217]. (2) Although prior research has primarily focused on the roles of exosomal miRNAs in renal inflammatory diseases, other biologically active molecules may also play significant roles in the function of EVs and require further investigation [148]. (3) EV isolation is challenging, with limitations in purification. Current methods, including superspeed centrifugation, size exclusion chromatography, ultrafiltration, immunocapture, precipitation, and microfluidic technology, often result in impurities and low yields, making the process cumbersome and time-consuming [218]. (4) Most clinical application studies of EVs have utilized traditional models like animal models. Incorporating human tissue models is essential for achieving clinical translation and ensuring the safety of drug delivery in humans [219]. (5) Integrating deep learning-based algorithms into therapeutic protocols is essential to realize precision medicine in the future, thus avoiding the adverse effects of “universal” protocols and customizing treatments for individual patients.

In response to the shortcomings of insufficient clinical production and low clinical translation efficiency of EV therapy, it is necessary to carry out engineering modifications of EVs (mainly including biological and chemical engineering techniques) to enhance targeting and treatment specificity to the injured site. Bioengineering introduces targeting motifs through gene fusion membrane binding proteins, and selectively sorts therapeutic cargo molecules into EVs or transfers them onto EVs by modifying their biogenic pathways [220,221,222,223]. In the process of bioengineering transformation, several points need to be noted [224]: (1) selecting the best engineering resources; (2) quality assurance and control in engineering; (3) EV recovery after bioengineering separation, purification, and storage. Chemical engineering refers to the process of adding substances such as antibodies, proteins, and small molecules through various chemical reactions to amplify the functionality and advantages of delivery systems [220,225]. There have been many studies on engineering EVs, such as the fusion of h-UC MSC EVs membrane with human neutrophil membrane to produce a hybrid vesicle called NEX. NEX is injected into the CI-AKI mouse model and can effectively act on the site of kidney injury; enhance antioxidant stress, anti-inflammatory, and anti-apoptotic [226]; and the expression of CD47+ on NEX membrane can inhibit the macrophage absorption of them [227]. EVs released by engineering MSCs mediate the transfer of miRNA-let7 to diseased kidneys and alleviate renal fibrosis by inhibiting fibrosis genes COL-4, MMP-9, α-smooth muscle actin (α-SMA), TGF-β 1, and their receptors [228]. In addition, changing the route of administration (intravenous/intrarenal) to improve the delivery of EVs to the kidneys and enhance EV management is also a way to improve performance. Obtaining deeper knowledge of EV stability and developing purification technologies to enrich specific EV populations will be key to achieving clinical translation in the future. Intelligent nanoEVs are also an emerging hotspot. When multivesicular vesicles are secreted together with the fused plasma membrane through the exocytosis of intracellular vesicles, intelligent nanoEVs are formed [229]. Previous studies have shown that intelligent nanoEVs can be secreted into extracellular regions to provide convenient conditions for examining bodily fluids such as urine. In the future, the prospects of intelligent nanoEVs as novel biomarkers for renal inflammatory diseases can be explored [230,231].

Further research into the multifaceted roles of EVs is needed to fully understand their therapeutic potential and to better utilize them for effective, safe, and targeted therapeutic interventions in inflammatory diseases of the kidney.

## Figures and Tables

**Table 1:** The role of EVs from different sources in AKI.

Origin	Product	Mechanism	Creature	References
MSCs	IDO	Regulation of macrophage polarization to M2-type promotes kidney self-repair in IRI	Mouse	[104]
Huc-MSCs	Unknown	Reduction in macrophage infiltration inhibits kidney inflammation, inhibits tubular cell apoptosis	Pig	[105]
Huc-MSCs	Unknown	Upregulation of miR-146b level reduces IRAK1 expression and inhibits NF-κB activity to suppress the secretion of inflammatory mediators by M1-type macrophages	Mouse	[46]
MSCs	CCR2	Reduce the concentration of free CCL2 to inhibit its recruitment and activation of macrophages	Mouse	[70]
MSCs	miR-100-5p	Targeting the FKBP5/AKT axis promotes M1 to M2 macrophage transformation and inhibits TEC apoptosis	Human being	[106]
USCs	miR-146a-5p	Targeted downregulation of IRAK-1 inhibits M1 macrophage polarization and attenuates HK-2 cell apoptosis	Human being	[107]
USCs	let-7b-5p	Targeting TLR4/NF-κB/STAT3/AKT promotes M2-type macrophage polarization	Human being	[108]
MSCs	miR-21	Targeting PDCD4 promotes M2 polarization	Mouse	[109]
hAECs	Multiple proteins	Reduction in macrophage infiltration to inhibit TNF-α production and ultimately suppress TNF α-induced inflammatory response and tubular cell apoptosis	Mouse	[110]
MSCs	miR-27b	Targeting the JMJD3/NF-κB/p6 axis to inhibit macrophage inflammation and reduce kidney injury	Mouse	[111]
Unknown	miR-30c-5p	Promotion of M1 macrophages transforming to M2 to attenuate kidney I/R injury	Rat	[112]
TECs	miR-20a-5p	Inhibition of macrophage infiltration prevents acute tubular injury	Mouse	[113]
ASCs	Unknown	Promotion of macrophage polarizing to M2-type to increase phagocytosis and inhibit TEC death	Human being	[114]
Macrophages (M2 > M1)	miR-93-5p	Regulation of miR-93/TXNIP signaling pathway inhibits TEC’s pyroptosis	Mouse	[79]
Macrophage	IL-10	Inhibition of mTOR signaling induces mitochondrial autophagy and promotes M2 polarization	Mouse	[115]
SCAP	Biologically active compound	Inhibition of oxidative stress, inflammation, and apoptosis to protect TECs	Rat	[89]

MSCs: mesenchymal stem cells; Huc-MSCs: human umbilical cord mesenchymal stem cells; USCs: urine-derived stem cells; hAECs: human amniotic epithelial cells; TECs: tubular epithelial cells; ASCs: adipose-derived stromal cells; SCAP: stem cell from the apical papilla; IDO: indoleamine 2,3-dioxygenase; CCR2: C-C motif chemokine receptor-2.

**Table 2:** The role of EVs from different sources in CKD.

Origin	Product	Mechanism	Creature	References
BMSCs	Unknown	Activate EP2 receptors and reduce the number of M1 and M2 macrophages to inhibit MMT process and alleviate renal fibrosis	UUO-Mouse	[170]
BMSCs	miR-204-5p	Regulation of the PI3K-AKT pathway affects autophagy, inhibits M1-type polarization, and promotes M2-type polarization	Mouse	[171]
Huc-MSCs	Unknown	Inhibition of kidney fibrosis by blocking the MMT process through the inhibition of ARNTL expression	UUO-Mouse	[172]
Huc-MSCs	miR-146a-5p	Targeting TRAF6 to promote M2-type macrophage polarization alleviates kidney impairment in DN	Human being	[173]
MSCs	miR-486-5p	Targeting PIK3R1 via the PI3K/Akt pathway promotes M2-type macrophage polarization and prevents diabetic nephropathy	Mouse	[174]
USCs	circRNA ATG7	Regulation of the SOCS1/STAT3 pathway by miR-4500 promotes macrophage polarization from M1 to M2 to inhibit DN progression	Human being	[175]
M2-type macrophage	miR-93-5p	Activation of autophagy by inhibiting DUSP1 expression ameliorates HG-induced podocyte damage	Mouse	[176]
M2-type macrophage	miR-25-3p	Targeting TLR4 attenuates podocyte apoptosis or upregulates ATXN3 expression to promote podocyte proliferation	Mouse	[162,177]
M1-type macrophage	miR-21a-5p	Inhibition of Tnpo1 expression in podocytes attenuates podocyte damage in HG	Mouse	[162]
Huc-MSCs	Unknown	Alleviation of podocyte injury in LN by promoting M2-type macrophage polarization	Human being	[178]
BMSCs	miR-16	Attenuation of lupus nephritis by regulating PDCD4 pathway to induce M2-type macrophage polarization	Mouse	[179]
BMSCs	miR-21	Alleviation of lupus nephritis by regulating PTEN pathway to induce M2-type macrophage polarization	Mouse	[179]
Unknown	LNA-anti-miR-150	Inhibition of M1 macrophage infiltration by targeting SOCS1 to attenuate LN-induced kidney injury	Mouse	[180]

BM-MSCs: bone marrow mesenchymal stem cell; HG: high glucose.

**Table 3:** EVs can serve as biomarkers for renal inflammatory diseases.

Kidney Disease	Urinary EV Product	Number Enrolled/Animal Model	Results	References
AKI	Fetuin-A	3 healthy volunteers 3 ICU patients with CI-AKI 3 ICU patients without AKI	Patients in the ICU with CI-AKI show a significant increase in Fetuin-A levels.	[186]
AKI	ATF3	S-AKI patients (n = 8) and healthy controls (n = 8)	Compared to healthy volunteers, S-AKI patients have higher levels of uATF3, which is negative in all the healthy volunteers.	[198]
AKI	Aquaporin-1	Renal ischemia/reperfusion rat model	Urinary vesicle aquaporin-1 abundance reduction emerges.	[199]
AKI	miR-16	Rat model of I/R-AKI	miR-16 expression is increased in UEs.	[200]
CKD	Ceruloplasmin	CKD (n = 15) and controls (n = 15)	Elevated urinary vesicle Ceruloplasmin was observed prior to proteinuria.	[201]
CKD	Osteoprotegerin	CKD (n = 14) and healthy controls (n = 4)	Urinary vesicle protein Osteoprotegerin levels are higher in patients with CKD than in healthy volunteers.	[202]
CKD	WT1	Type-1 diabetes mellitus patients (n = 48) patients and healthy controls (n = 25)	The WT1 protein is predominantly present in UE of diabetic patients, with its expression levels increasing as kidney function declines.	[191,194]
CKD	CD63	Microalbuminuria-stage DN patients (n = 62) and controls (n = 29)	During the early stages of DN, the content of EVs containing CD63 in urine significantly increased.	[203]
CKD	E-cadherin	27 prevalent case-patients with posterior urethral valves and 20 age-matched controls	Patients excrete significantly lower levels of E-cadherin.	[204]
CKD	TGF-B1	27 prevalent case-patients with posterior urethral valves and 20 age-matched controls	Patients excrete significantly higher levels of TGF-B1.	[204]
CKD	N-cadherin	27 prevalent case-patients with posterior urethral valves and 20 age-matched controls	Patients excrete significantly lower levels of N-cadherin.	[204]
CKD	L1CAM	27 prevalent case-patients with posterior urethral valves and 20 age-matched controls	Patients excrete significantly higher levels of L1CAM.	[204]
CKD	miR-192	80 patients diagnosed with Type-2 diabetes (30 normoal buminuria, 30 microalbuminuria, and 20 macroalbuminuria) and 10 healthy controls	miR-192 is better than miR-194 and miR-215 in identifying DN in patients with normoal buminuria or microalbuminuria, which indicated that uEVs miR-192 might be able to diagnose DN earlier.	[205]
CKD	miR-15a-5p	40 patients with type-2 diabetes mellitus (T2DM) patients, 20 patients with normoal buminuria, and 20 patients with macroalbuminuria	miR-15a-5p is downregulated in DN patients compared to T2DM patients.	[206]
CKD	miR-29c	45 LN patients and 20 healthy/LN (n = 32), nonlupus CKD patients (n = 15) and healthy controls (n = 20)	In patients with LN, urinary vesicle miR-29c may serve as a potential biomarker for predicting disease progression.	[197,207]
CKD	miR-200b/miR-200	38 CKD patients with different degrees of renal fibrosis and in 12 normal individuals/32 CKD patients and 7 controls	The level of miR-200b in the CKD group is lower and negatively correlated with fibrosis	[208]

## Data Availability

Not applicable.
